# Body mass index and infection risks in people with and without type 2 diabetes: A cohort study using electronic health records

**DOI:** 10.1038/s41366-025-01828-z

**Published:** 2025-07-18

**Authors:** Iain M. Carey, Tess Harris, Umar A. R. Chaudhry, Stephen DeWilde, Elizabeth S. Limb, Liza Bowen, Selma Audi, Derek G. Cook, Peter H. Whincup, Naveed Sattar, Arshia Panahloo, Julia A. Critchley

**Affiliations:** 1 Population Health Research Institute, St George’s School of Health and Medical Sciences, City St George’s, University of London, London, SW17 0RE UK; 2 School of Cardiovascular and Metabolic Health, BHF Glasgow Cardiovascular Research Centre, University of Glasgow, 126 University Place, Glasgow, G12 8TA Scotland; 3 St George’s University Hospitals NHS Foundation Trust, Blackshaw Road, Tooting, London, SW17 0QT UK

**Keywords:** Risk factors, Disease prevention, Epidemiology

## Abstract

**Background:**

Observational studies have found U-shaped associations between body mass index (BMI) and infections. While people with type 2 diabetes (T2D) are generally more likely to live with obesity and be at higher risk of infections, it is unknown whether BMI has the same impact on infection risk in people with and without diabetes, and whether this varies by type of infection.

**Methods:**

516,935 people with T2D and 751,909 people without diabetes, aged 18–90 and alive on 1/1/2015 with BMI measured during 2011–14 matched on age, sex and ethnicity in the Clinical Practice Research Datalink. Infections during 2015–19 were collated from primary care and linked hospitalisation records. Poisson regression estimated incidence rate ratios (IRR) for infection, across 12 BMI categories (from ≤19 kg/m^2^ to >48 kgm^2^) using a reference group of >24–≤26 kg/m^2^. Separate models for T2D and non-diabetes were used, adjusting for age, sex, ethnicity, deprivation, smoking and co-morbidity. Additional analyses explored associations by common infection types.

**Results:**

People with T2D were at higher infection risk than people without diabetes at all BMI levels, however the pattern observed at different BMI levels was similar in both groups (e.g. BMI >48 compared to BMI >24–≤26, T2D IRR = 2.35, 95%CI 2.26–2.44, non-diabetes IRR = 2.52, 95%CI 2.30–2.75 for hospitalisation-related infections). Hospitalisation-related infections showed a U-shaped association with BMI not explained by age, smoking or co-morbidity, whereas primary care infections were predominately associated with higher BMI levels only. Cellulitis showed the strongest trends in relation to high BMI levels, whilst lower respiratory tract infection and sepsis were related to both high and low BMI levels.

**Conclusions:**

Infection risks are consistently higher for people with T2D but the association with increasing levels of BMI appears similar both in patients with and without diabetes. Additionally, being underweight is also associated with increased risk of infections requiring hospitalisation.

## Background

The relationship between obesity and infection was frequently highlighted during the COVID-19 pandemic [1], with numerous studies observing strong associations in adults of all ages [2]. With obesity prevalence rising [3], the potential public health importance of this association has been increasing, though the evidence base for many infections was limited [4]. Recently, there is more evidence emerging from observational studies that obesity, estimated from body mass index (BMI), is associated with infection risk [5–8]. Further, it has also been observed that for some infections, such as pneumonia, the relationship is U-shaped, such that a lower BMI as well as a higher one is also significantly related to higher infection risk [9–11]. However, the shape of the association with BMI may not be consistent across different infection types, with skin and urinary tract infections not necessarily being related to being underweight [9].

Obesity is also strongly associated with type 2 diabetes (T2D) [12], which has itself been shown to be related to greater risk of future infections [13]. However, it is not known if T2D mediates the association between obesity and infection, and whether the shape of the association with BMI is similar between people with T2D and people without diabetes. Recent evidence suggests that infection risk among people with T2D is heterogenous, with abdominal obesity being highly influential [14]. Given the high global prevalence of both T2D and obesity [15], with the recent emergence of novel weight loss therapies [16, 17], a better understanding of the interaction between BMI, diabetes and infection would be desirable and informative for patients, clinicians and policy-makers.

This study investigated whether (i) the relationship between BMI and infection risk is similar between people with T2D and people without diabetes, (ii) the magnitude and shape of the associations differs by age, sex and infection type. By utilising a large English cohort of electronic patient records of people with T2D, the study summarises findings on the associations with different infections at more extreme levels of BMI than have been previously reported. It also makes use of a matched cohort without diabetes who have been matched by age, sex and ethnicity to compare the associations across the same range of BMI values.

## Methods

### Data sources

CPRD (Clinical Practice Research Datalink) is a UK primary care database jointly sponsored by the Medicines and Healthcare products Regulatory Agency and the National Institute for Health and Care Research [18]. It provides a pseudonymised longitudinal medical record for all registered patients (>99% of the UK population are registered with a General Practitioner), with diagnoses and other clinical information recorded using Read Codes. This study used an extract from the CPRD Aurum database that included approximately 16 million currently registered patients from 1447 general practices (England only). Over 90% of contributing practices in Aurum have consented to their data being linked to external sources, and researchers have no access to geographical identifiers such as residential postcode [19]. These data sources include the ONS (Office for National Statistics) mortality data which includes cause of death and the Index of Multiple Deprivation (IMD), a composite small-area (approximately 1500 people) measure used in England for allocation of resources, which provides a good proxy for individual socio-economic deprivation.

### Study design and participants

The study was a matched cohort design comparing people with diabetes to a group without diabetes, described previously [13]. Briefly, all patients aged 18 to 90 with a Read code for diabetes who were active in CPRD on 1st January 2015 and had been registered for at least one year were first identified, and classified into Type 1, Type 2 or unknown based on their diagnosis codes and anti-diabetes medication. For this analysis, the main focus was on 525,812 people with T2D (Fig. S1) who had been randomly matched to at least one person without known prediabetes or diabetes, of the same age, sex and ethnicity (White, South Asian, Black, mixed/other or not recorded). A maximum of two people without diabetes was selected for each person with T2D resulting initially in a total of 1,008,898 people (with 94% of match-sets having two comparators, 6% having one).

### Body mass index

For each person in the study, the last recorded BMI during 2011–14 was used. If only a weight measurement was present, a BMI was manually estimated if a height measurement was available anywhere in their record (using the closest height measurement to 1/1/2015 when there were multiple measurements). Only BMI measurements between 12 and 85 kg/m^2^ were accepted as plausible. To compare BMI between people with T2D and without diabetes, and explore the non-linearity at fine detail, 12 BMI categories were created (≤19, >19–≤22, >22–≤24, >24–≤26, >26–≤28, >28–≤30, >30–≤32, >32–≤35, >35–≤39, >39–≤43, >43–≤48, >48) with sufficient numbers in the extreme categories for both the T2D and non-diabetes groups. Thus, ≤19 was chosen as the extreme underweight category rather than the conventional 18.5 because there were too few people with T2D with BMIs in this range. The BMI category >24–≤26, was chosen a priori as the reference category for both within group comparisons. This category has an approximate mean of 25 kg/m^2^, which was the value with lowest infection risk in a recent large population study from Denmark [9]. While over 98% of people with T2D had a BMI measured during 2011–14 (*n* = 516,935), this was true for only 75% of people without diabetes (*n* = 751,909) (Fig. S1). Although this resulted in some people with T2D no longer having a matched person without diabetes, since they are not directly being compared to each other here, these groups were used in the main analysis to maximise the total number. However, a sensitivity analysis considered only match-sets with 1:1 matching (*n* = 471,474 people with T2D).

### Infection outcomes and covariates

Infections were collated using an extensive list of Read codes (primary care) and ICD-10 codes (hospital data), previously described [13]. Briefly, both data sources were searched electronically over a 5-year period (2015–19) for the following: (i) any infection with a prescription in primary care for an antibiotic, antifungal or antiviral within ±14 days of the diagnosis; (ii) any new hospital episode where an infection was the primary diagnosis. In the UK, hospital data is organised into finished consultant episodes and assigned a primary diagnosis [20]. Subsequent episodes can be assigned to the same admission, with a different primary diagnosis e.g., a hospital acquired infection. For each summary group, only one event was counted within a 90-day period, with multiple codes assumed to be the same event. A further analysis considered type of infection from either source, using the 8 most common groups - cellulitis, gastrointestinal tract (GIT), genitourinary (GUI), lower respiratory tract (LRT), mycoses, sepsis, skin (other) and upper respiratory tract (URT). Read code lists for the infection groups are available in the repository 10.24376/rd.sgul.21565557.v1.

Information was also extracted on patient smoking history, and co-morbidities as of 1st January 2015. We selected 12 chronic conditions routinely collected as part of the Quality and Outcomes Framework (QOF), a UK wide system for performance management and payment of GPs in primary care [21]. These were atrial fibrillation, cancer, chronic obstructive pulmonary disease, coronary heart disease, chronic kidney disease, dementia, epilepsy, heart failure, hypertension, peripheral vascular disease, serious mental Illness and stroke.

### Statistical methods

Age-sex standardised incidence rates for each infection outcome were estimated, using the distribution of patients with T2D as the standard population. Poisson regression compared infection rates during follow-up for patients with T2D and without diabetes in separate models, with an offset fitted for total days of follow-up time in the study (SAS version 9.4). The 12 BMI categories were fitted, with Incidence Rate Ratios (IRRs) estimating infection risk compared to the >24–≤26 reference category. These were initially fitted adjusting for age (10-year age groups) and sex. Further adjustment added ethnicity (5 categories including not recorded), IMD (quintile), smoking and a count of co-morbidities. For each model an estimated attributable risk fraction (AF) was estimated [22], which assume the associations are causal and that all patients are shifted to the reference category (BMI >24–≤26). All analyses were stratified by sex and age group (18–50, 51–70, 71–90). The following sensitivity analyses were carried out: (i) restricting the analysis to never smokers and those without any co-morbidity, (ii) repeating the main analysis but only using 1:1 match-sets.

## Results

### Summary of BMI

The mean age of the 516,935 people with T2D in 2015 was 66.3 years (SD = 12.9) with 56% of them males (Table 1); similar to the non-diabetes group (66.8 years, 56% respectively). People with T2D were more likely to be living in more deprived areas and have more co-morbidities than people without diabetes. The mean BMI was substantially higher among people with T2D compared to the non-diabetes group (31.0 vs. 27.2 kg/m^2^). The distributions of BMI was more spread out among people with T2D at all ages (Fig. S2), with approximately 50% of people with T2D had a recorded BMI of 30 or greater compared to 24% of the non-diabetes group. In both groups there was a clear trend of descending age with increasing BMI (Fig. S3), though the difference in age between the extreme categories of BMI was greater in people with T2D (approximately 16 years vs. 10 years for people without diabetes).

**Table 1:** Baseline characteristics of people with and without type 2 diabetes with a BMI recorded in last 5 years.

			People with T2D (*n* = 516,935)		Non-diabetes (*n* = 751,909)	
Follow-up (in years)	All	mean, SD	4.2	1.4	4.4	1.3
Sex	Females	*n*, %	224 720	44.1%	430 819	44.1%
	Males	*n*, %	284 683	55.9%	545 612	55.9%
Age (in 2015)	All	mean, SD	66.3	12.9	66.8	12.7
	18–50	*n*, %	64 931	12.6%	85 848	11.4%
	51–70	*n*, %	241 058	46.6%	347 435	46.2%
	71–90	*n*, %	210 946	40.8%	318 626	42.4%
Body Mass Index (kg/m^2^)	All	mean, SD	31.0	6.4	27.2	5.1
	≤19	*n*, %	4 002	0.8%	19 955	2.7%
	>19–≤22	*n*, %	19 046	3.7%	77 819	10.4%
	>22–≤24	*n*, %	33 779	6.5%	104 802	13.9%
	>24–≤26	*n*, %	55 828	10.8%	135 926	18.1%
	>26–≤28	*n*, %	70 961	13.7%	130 503	17.4%
	>28–≤30	*n*, %	73 223	14.2%	101 638	13.5%
	>30–≤32	*n*, %	67 946	13.1%	70 402	9.4%
	>32–≤35	*n*, %	76 330	14.8%	58 857	7.8%
	>35–≤39	*n*, %	61 284	11.9%	33 331	4.4%
	>39–≤43	*n*, %	29 845	5.8%	11 877	1.6%
	>43–≤48	*n*, %	15 934	3.1%	4 793	0.6%
	>48	*n*, %	8 757	1.7%	2 006	0.3%
Ethnicity	South Asian	*n*, %	53 097	10.3%	65 662	8.7%
	Black	*n*, %	22 069	4.3%	30 280	4.0%
	Mixed	*n*, %	29 905	5.8%	43 463	5.8%
	White	*n*, %	363 561	70.3%	563 588	75.0%
	Missing	*n*, %	48 303	9.3%	48 916	6.5%
Index of Multiple Deprivation*	1 (least)	*n*, %	85 809	16.6%	168 240	22.4%
	2	*n*, %	95 817	18.5%	163 008	21.7%
	3	*n*, %	99 751	19.3%	147 526	19.6%
	4	*n*, %	114 640	22.2%	144 485	19.2%
	5 (most)	*n*, %	120 551	23.3%	128 084	17.0%
	Missing	*n*, %	367	0.1%	566	0.1%
Smoking	Never	*n*, %	193 200	37.4%	329 071	43.8%
	Ex	*n*, %	250 023	48.4%	322 738	42.9%
	Current	*n*, %	73 648	14.3%	99 635	13.3%
	Not recorded	*n*, %	64	0.0%	465	0.1%
Number of co-morbidities‡	0	*n*, %	122 204	23.6%	338 491	45.0%
	1-2	*n*, %	304 336	58.9%	341 208	45.4%
	>2	*n*, %	90 395	17.5%	72 210	9.6%

* - A composite small-area (approximately 1500 people) measure used in England for allocation of resources [40]. ‡ - Count of the following: Atrial fibrillation, cancer, chronic obstructive pulmonary disease, coronary heart disease, chronic kidney disease, dementia, epilepsy, heart failure, hypertension, peripheral vascular disease, serious mental Illness (e.g. psychosis, schizophrenia & bipolar affective disorder), stroke/TIA.

### Incidence of overall infection by BMI

**Fig. 1:**
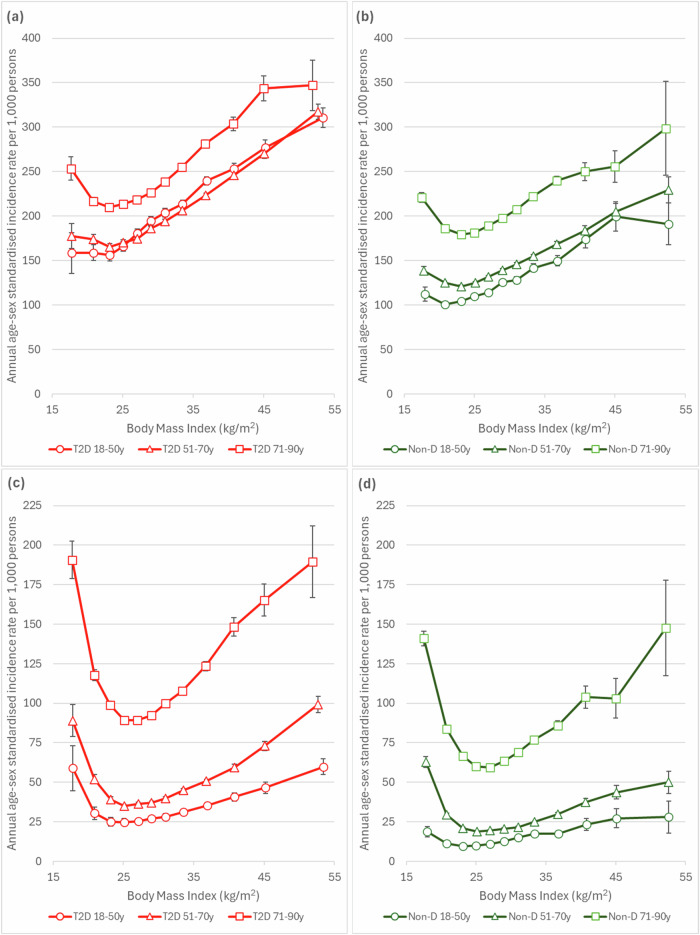
Incidence rates for infections during 2015–2019 across BMI categories by age group in people with and without type 2 diabetes (T2D) separately. **a** Primary care infections (T2D, *n*=516,935). **b** Primary care infections (non-diabetes, *n*=751,909). **c** Hospitalisation-related infections (T2D, *n*=516,935). **d** Hospitalisation-related infections (non-diabetes, *n*=751,909). Incidence rates were age-standardised to T2D population. Error bars show 95% confidence intervals.

Figure 1 summarises age-sex standardised incidence rates for the primary care and hospitalisation related infections in people with T2D and without diabetes separately by different age groups. People with T2D are at a higher risk for infections, both from primary care and those requiring hospitalisation, than people without diabetes at all BMI levels. The lowest incidence rates are observed at approximately 23 kg/m^2^ for primary care and 25 kg/m^2^ for hospitalisation related infections. Both outcomes show a consistent positive association with increasing levels of BMI, which are similar in people with T2D and without diabetes. For hospitalisation related infections there is also strong evidence of a U-shaped association with BMI at all ages, which is seen in both groups. However, for primary care infections, the higher incidence of infection at the lowest BMI level is much less apparent. Stratifying the overall analysis by sex showed similar trends with BMI, with women having consistently higher primary care infection rates (Fig. S4).

### Relative risks of infection with elevated or low BMI

Table 2 compares adjusted incidence rates for the two summary infection groups, in people with T2D and without diabetes separately, using a BMI of >24–≤26 as a reference category, overall and by age group. The elevated relative risks at extreme high and low levels of BMI are much more apparent for hospitalisation related infections than for those seen in primary care. People in the highest category (BMI > 48) had a more than doubling of this risk compared to those with a BMI of >24– ≤ 26 (T2D IRR = 2.35, 95%CI 2.26–2.44, non-diabetes IRR = 2.52, 95%CI 2.30–2.75), and this pattern was seen at all ages. People in the most underweight category (BMI ≤ 19) had elevated risks of hospitalisation related infections (T2D IRR = 1.92 95%CI 1.83–2.02, non-diabetes IRR = 2.11 95%CI 2.05–2.17) compared to a BMI of >24–≤26. For primary care infections, the small increase in risk among underweight was only observed among ages 71–90 (Fig. S5). When the analysis was stratified by sex (Table S1), men tended to have higher IRRs at the extreme BMI categories in both people with T2D and without diabetes. Attributable risk estimates (Table S2) suggested that the combined contribution of under- and over- weight on primary care infections was greater in people with T2D (11.4%) compared to people without diabetes (5.9%), but for hospitalisation related infections it was more similar due to a greater proportion of people without diabetes being underweight (11.5% vs. 10.0% respectively).

**Table 2:** Adjusted incidence rate ratios for infections during 2015–19 by BMI in people with T2D and without diabetes.

Outcome/BMI	Type 2 Diabetes (*n* = 516,935)				Non-Diabetes (*n* = 751,909)			
	All	Ages 18–50	Ages 51–70	Ages 71–90	All	Ages 18-50	Ages 51–70	Ages 71–90
	IRR (95% CI)	IRR (95% CI)	IRR (95% CI)	IRR (95% CI)	IRR (95% CI)	IRR (95%CI)	IRR (95%CI)	IRR (95%CI)
Primary Care								
≤19	1.08 (1.04–1.12)	0.89 (0.77–1.02)	1.00 (0.92–1.08)	1.16 (1.11–1.21)	1.11 (1.09–1.13)	0.94 (0.88–1.00)	1.04 (1.00–1.07)	1.19 (1.16–1.22)
>19–≤22	1.00 (0.98–1.01)	0.94 (0.89–1.01)	1.00 (0.97–1.03)	1.01 (0.99–1.04)	1.00 (0.99–1.01)	0.89 (0.86–0.92)	0.98 (0.96–1.00)	1.04 (1.02–1.06)
>22–≤24	0.97 (0.95–0.98)	0.93 (0.89–0.99)	0.96 (0.94–0.99)	0.98 (0.96–1.00)	0.98 (0.97–0.99)	0.94 (0.91–0.97)	0.97 (0.95–0.98)	1.00 (0.99–1.01)
>24–≤26	1 (Reference)	1 (Reference)	1 (Reference)	1 (Reference)	1 (Reference)	1 (Reference)	1 (Reference)	1 (Reference)
>26–≤28	1.02 (1.01–1.04)	1.08 (1.04–1.13)	1.03 (1.01–1.05)	1.01 (1.00–1.03)	1.04 (1.03–1.05)	1.03 (1.00–1.07)	1.05 (1.03–1.06)	1.04 (1.02–1.05)
>28–≤30	1.08 (1.06–1.09)	1.16 (1.11–1.21)	1.10 (1.08–1.12)	1.04 (1.03–1.06)	1.09 (1.08–1.10)	1.14 (1.10–1.18)	1.10 (1.08–1.12)	1.08 (1.06–1.09)
>30–≤32	1.13 (1.11–1.14)	1.21 (1.16–1.26)	1.15 (1.13–1.17)	1.10 (1.08–1.12)	1.13 (1.12–1.14)	1.15 (1.11–1.20)	1.15 (1.13–1.17)	1.12 (1.10–1.14)
>32–≤35	1.20 (1.18–1.21)	1.27 (1.22–1.32)	1.23 (1.20–1.25)	1.17 (1.15–1.19)	1.20 (1.19–1.21)	1.28 (1.23–1.32)	1.21 (1.19–1.23)	1.19 (1.17–1.20)
>35–≤39	1.30 (1.29–1.32)	1.41 (1.36–1.46)	1.32 (1.30–1.35)	1.28 (1.25–1.30)	1.29 (1.28–1.31)	1.34 (1.29–1.40)	1.30 (1.28–1.33)	1.27 (1.24–1.30)
>39–≤43	1.41 (1.39–1.43)	1.47 (1.41–1.53)	1.44 (1.41–1.47)	1.38 (1.34–1.41)	1.39 (1.36–1.41)	1.57 (1.48–1.66)	1.38 (1.35–1.42)	1.31 (1.27–1.36)
>43–≤48	1.57 (1.54–1.59)	1.60 (1.54–1.68)	1.58 (1.54–1.62)	1.55 (1.50–1.60)	1.51 (1.47–1.56)	1.65 (1.53–1.77)	1.49 (1.43–1.55)	1.45 (1.37–1.53)
>48–	1.75 (1.71–1.78)	1.76 (1.68–1.84)	1.77 (1.72–1.82)	1.65 (1.57–1.74)	1.68 (1.61–1.75)	1.66 (1.50–1.84)	1.69 (1.60–1.78)	1.55 (1.40–1.71)
Hospitalisations								
≤19	1.92 (1.83–2.02)	2.02 (1.59–2.57)	2.29 (2.05–2.57)	1.86 (1.75–1.96)	2.11 (2.05–2.17)	1.53 (1.29–1.83)	2.54 (2.40–2.69)	2.02 (1.96–2.09)
>19–≤22	1.31 (1.28–1.35)	1.18 (1.01–1.37)	1.44 (1.35–1.53)	1.29 (1.25–1.34)	1.36 (1.34–1.39)	1.03 (0.92–1.16)	1.41 (1.35–1.47)	1.37 (1.33–1.40)
>22–≤24	1.10 (1.08–1.13)	1.01 (0.88–1.16)	1.13 (1.07–1.19)	1.10 (1.07–1.14)	1.11 (1.09–1.13)	0.95 (0.85–1.07)	1.10 (1.06–1.15)	1.12 (1.09–1.15)
>24–≤26	1 (Reference)	1 (Reference)	1 (Reference)	1 (Reference)	1 (Reference)	1 (Reference)	1 (Reference)	1 (Reference)
>26–≤28	0.99 (0.97–1.01)	1.00 (0.90–1.12)	1.02 (0.97–1.06)	0.98 (0.96–1.01)	0.98 (0.96–1.00)	1.07 (0.96–1.19)	1.01 (0.97–1.05)	0.97 (0.95–0.99)
>28–≤30	1.00 (0.98–1.03)	1.04 (0.93–1.16)	1.02 (0.98–1.06)	1.00 (0.98–1.03)	1.03 (1.01–1.05)	1.23 (1.10–1.38)	1.05 (1.01–1.09)	1.02 (0.99–1.04)
>30–≤32	1.07 (1.04–1.09)	1.05 (0.95–1.17)	1.07 (1.03–1.12)	1.08 (1.05–1.11)	1.09 (1.07–1.12)	1.42 (1.26–1.59)	1.08 (1.04–1.13)	1.09 (1.06–1.12)
>32–≤35	1.15 (1.13–1.18)	1.15 (1.04–1.27)	1.19 (1.14–1.24)	1.14 (1.11–1.17)	1.22 (1.19–1.24)	1.60 (1.43–1.80)	1.22 (1.17–1.28)	1.20 (1.16–1.23)
>35–≤39	1.30 (1.27–1.33)	1.25 (1.13–1.38)	1.32 (1.26–1.37)	1.30 (1.27–1.34)	1.37 (1.34–1.41)	1.63 (1.43–1.86)	1.43 (1.36–1.50)	1.33 (1.28–1.38)
>39–≤43	1.51 (1.47–1.56)	1.40 (1.26–1.55)	1.51 (1.44–1.58)	1.54 (1.48–1.60)	1.66 (1.60–1.74)	2.00 (1.69–2.36)	1.73 (1.62–1.85)	1.57 (1.48–1.66)
>43–≤48	1.77 (1.72–1.83)	1.51 (1.35–1.69)	1.78 (1.69–1.87)	1.80 (1.71–1.90)	1.93 (1.82–2.05)	2.31 (1.87–2.85)	2.00 (1.83–2.19)	1.78 (1.63–1.95)
>48–	2.35 (2.26–2.44)	1.94 (1.73–2.18)	2.39 (2.26–2.52)	2.32 (2.16–2.50)	2.52 (2.30–2.75)	2.12 (1.58–2.84)	2.32 (2.05–2.63)	2.88 (2.50–3.31)

Separate models for T2D and non–diabetes. IRRs adjust for age, sex, ethnicity, deprivation, smoking and co–morbidity count.

Sensitivity analyses adjusting only for age and sex or restricting to 1:1 match-sets produced similar IRRs to the main analysis (Table S3). When the overall analysis was restricted to never smokers, those without co-morbidity, or both, the IRRs observed with high BMI levels were unchanged, and the association between underweight BMI levels and hospitalisation related infections remained similar (Table S4).

### Risks with specific infection groups

**Fig. 2:**
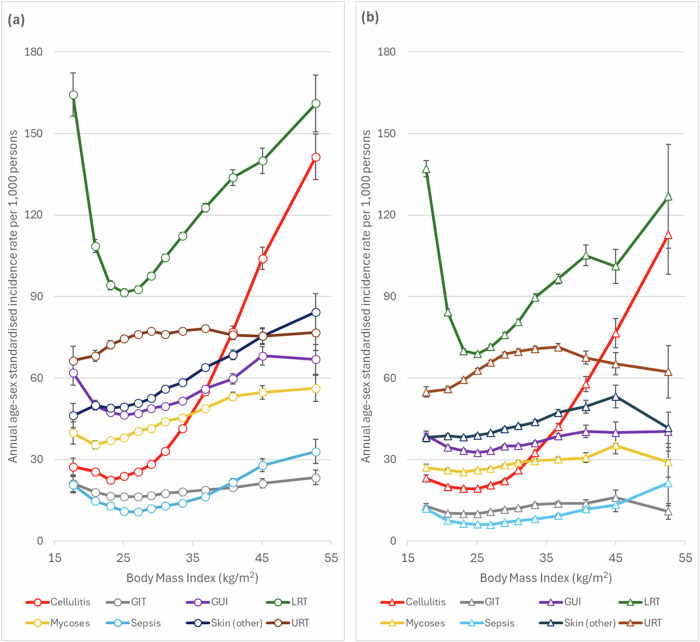
Incidence rates by infection type during 2015–2019 across BMI categories in people with and without type 2 diabetes (T2D) separately. **a** T2D (*n*=516,935). **b** Non-diabetes (*n*=751,909). Incidence rates were age-standardised to T2D population. Error bars show 95% confidence intervals.

Figure 2 summarises age-sex standardised incidence rates by infection type in people with T2D and without diabetes. The pattern of infection incidence with BMI varied greatly by infection type but were comparable between people with and without T2D (Fig. 2). Cellulitis had a distinctive J-shape, while lower respiratory tract was the most U-shaped with similar raised incidence at both the lowest and highest BMI levels. In the adjusted regression models (Table 3), people in the highest category (BMI > 48) had >6 times risk of cellulitis compared to those with a BMI of >24–≤26 (T2D IRR = 6.19, 95%CI 5.94–6.45, non-diabetes IRR = 6.43, 95%CI 5.98–6.92) and >3 times risk of sepsis (T2D IRR = 3.23, 95%CI 2.99–3.50, non-diabetes IRR = 3.30, 95% CI 2.71–4.03), with smaller increases in risk observed for all other types. For people in the underweight category (BMI ≤ 19), the risks compared to those with a BMI of >24–≤26 were approximately 1.6 times for both LRT infections and sepsis (IRR = 1.60, 95% CI 1.53–1.67 and IRR = 1.63, 95% CI 1.44–1.84 for people with T2D respectively), whereas there was no evidence of an increase in risk at low BMI for skin or URT infections.

**Table 3:** Adjusted incidence rate ratios for infections during 2015–19 by BMI in people with T2D and without diabetes, by type.

Group/BMI	Cellulitis	GIT	GUI	LRT	Mycoses	Sepsis	Skin (other)	URT
	IRR (95% CI)	IRR (95% CI)	IRR (95% CI)	IRR (95% CI)	IRR (95% CI)	IRR (95% CI)	IRR (95% CI)	IRR (95% CI)
T2 Diabetes (n=516,935)								
≤19	1.15 (1.04–1.27)	1.20 (1.07–1.36)	1.20 (1.12–1.28)	1.60 (1.53–1.67)	1.01 (0.92–1.11)	1.63 (1.44–1.84)	0.90 (0.82–0.98)	0.88 (0.82–0.95)
>19–≤22	1.09 (1.04–1.15)	1.07 (1.00–1.14)	1.07 (1.04–1.11)	1.14 (1.11–1.17)	0.89 (0.85–0.93)	1.31 (1.22–1.40)	0.99 (0.95–1.02)	0.89 (0.86–0.92)
>22–≤24	0.96 (0.92–1.00)	1.01 (0.96–1.06)	1.03 (1.00–1.06)	1.02 (1.00–1.04)	0.95 (0.92–0.99)	1.16 (1.10–1.23)	0.98 (0.95–1.01)	0.95 (0.93–0.98)
>24–≤26	1 (Reference)	1 (Reference)	1 (Reference)	1 (Reference)	1 (Reference)	1 (Reference)	1 (Reference)	1 (Reference)
>26–≤28	1.04 (1.01–1.08)	1.00 (0.95–1.04)	1.00 (0.97–1.02)	1.00 (0.98–1.01)	1.07 (1.04–1.10)	0.96 (0.91–1.01)	1.02 (1.00–1.05)	1.04 (1.02–1.06)
>28–≤30	1.13 (1.09–1.17)	1.01 (0.97–1.05)	1.02 (0.99–1.04)	1.04 (1.02–1.06)	1.11 (1.08–1.14)	1.05 (1.00–1.10)	1.06 (1.03–1.08)	1.07 (1.05–1.10)
>30–≤32	1.31 (1.26–1.35)	1.07 (1.02–1.11)	1.02 (1.00–1.05)	1.10 (1.08–1.12)	1.19 (1.16–1.23)	1.11 (1.06–1.17)	1.12 (1.09–1.15)	1.08 (1.06–1.10)
>32–≤35	1.59 (1.54–1.64)	1.09 (1.04–1.13)	1.05 (1.03–1.08)	1.18 (1.16–1.20)	1.24 (1.21–1.27)	1.17 (1.11–1.23)	1.17 (1.14–1.20)	1.12 (1.10–1.14)
>35–≤39	2.08 (2.01–2.15)	1.13 (1.08–1.18)	1.13 (1.10–1.16)	1.27 (1.25–1.29)	1.35 (1.32–1.39)	1.35 (1.28–1.42)	1.28 (1.25–1.31)	1.16 (1.14–1.18)
>39–≤43	2.88 (2.78–2.98)	1.19 (1.13–1.25)	1.17 (1.13–1.20)	1.38 (1.35–1.41)	1.49 (1.44–1.54)	1.71 (1.61–1.81)	1.40 (1.36–1.44)	1.16 (1.13–1.19)
>43–≤48	3.90 (3.76–4.05)	1.23 (1.16–1.31)	1.30 (1.25–1.35)	1.50 (1.46–1.54)	1.55 (1.50–1.61)	2.14 (2.00–2.29)	1.51 (1.46–1.56)	1.20 (1.17–1.24)
>48–	6.19 (5.94–6.45)	1.37 (1.27–1.48)	1.41 (1.35–1.48)	1.72 (1.67–1.78)	1.59 (1.52–1.66)	3.23 (2.99–3.50)	1.71 (1.64–1.77)	1.18 (1.13–1.22)
Non–Diabetes (n=751,909)								
19	1.23 (1.18–1.30)	1.19 (1.11–1.27)	1.13 (1.09–1.17)	1.65 (1.62–1.69)	1.01 (0.97–1.06)	1.76 (1.63–1.89)	0.97 (0.93–1.01)	0.85 (0.82–0.88)
>19–≤22	1.08 (1.05–1.11)	1.00 (0.95–1.04)	1.08 (1.05–1.10)	1.17 (1.15–1.19)	0.99 (0.96–1.01)	1.19 (1.13–1.26)	0.99 (0.97–1.01)	0.89 (0.88–0.91)
>22–≤24	1.02 (0.99–1.05)	1.00 (0.97–1.04)	1.04 (1.02–1.07)	1.02 (1.00–1.03)	0.97 (0.94–0.99)	1.06 (1.01–1.11)	0.98 (0.96–1.00)	0.95 (0.93–0.96)
>24–≤26	1 (Reference)	1 (Reference)	1 (Reference)	1 (Reference)	1 (Reference)	1 (Reference)	1 (Reference)	1 (Reference)
>26–≤28	1.06 (1.03–1.09)	1.07 (1.03–1.11)	1.00 (0.98–1.03)	1.02 (1.01–1.04)	1.02 (0.99–1.04)	0.96 (0.91–1.00)	1.02 (1.00–1.04)	1.04 (1.03–1.06)
>28–≤30	1.14 (1.11–1.17)	1.13 (1.09–1.18)	1.04 (1.02–1.06)	1.07 (1.05–1.08)	1.06 (1.03–1.08)	1.05 (1.00–1.11)	1.05 (1.03–1.08)	1.08 (1.07–1.10)
>30–≤32	1.32 (1.28–1.36)	1.17 (1.12–1.22)	1.02 (1.00–1.05)	1.13 (1.11–1.15)	1.10 (1.07–1.13)	1.13 (1.07–1.19)	1.08 (1.06–1.10)	1.10 (1.08–1.12)
>32–≤35	1.62 (1.58–1.67)	1.29 (1.24–1.35)	1.04 (1.02–1.07)	1.24 (1.22–1.26)	1.12 (1.09–1.15)	1.21 (1.14–1.28)	1.12 (1.10–1.15)	1.12 (1.10–1.14)
>35–≤39	2.05 (1.98–2.11)	1.31 (1.24–1.38)	1.09 (1.06–1.12)	1.33 (1.30–1.36)	1.16 (1.12–1.20)	1.38 (1.29–1.47)	1.22 (1.19–1.25)	1.14 (1.12–1.17)
>39–≤43	2.84 (2.72–2.97)	1.28 (1.18–1.38)	1.12 (1.06–1.17)	1.45 (1.40–1.49)	1.21 (1.15–1.27)	1.70 (1.53–1.88)	1.24 (1.19–1.29)	1.14 (1.11–1.18)
>43–≤48	3.98 (3.76–4.21)	1.51 (1.35–1.69)	1.12 (1.04–1.20)	1.49 (1.42–1.56)	1.42 (1.32–1.53)	2.17 (1.88–2.51)	1.40 (1.32–1.48)	1.12 (1.07–1.18)
>48–	6.43 (5.98–6.92)	1.31 (1.09–1.58)	1.05 (0.94–1.19)	1.79 (1.67–1.92)	1.19 (1.06–1.35)	3.30 (2.71–4.03)	1.40 (1.28–1.54)	1.08 (1.00–1.16)

Separate models for T2D and non–diabetes. IRRs adjust for age, sex, ethnicity, deprivation, smoking and co-morbidity count.

## Discussion

This study used large electronic health databases to describe the associations between body mass index and infection risk, both within a cohort of people with T2D, and a comparison group without diabetes. Despite people with T2D having higher BMI levels, and being at greater risk of infections overall, it found that the relationship between BMI and infection was similar in people with and without diabetes. People classed as having obesity based on their BMI, regardless of whether they had T2D or not, were at a consistently higher risk of acquiring an infection over a 5-year period compared to persons of normal weight, particularly cellulitis. Additionally, very low BMI levels were also associated with a higher risk of infections requiring hospitalisation, particularly lower respiratory tract infections and sepsis, though not with infections treated in primary care.

### Strengths and limitations

One of the main strengths of the present analysis is the large sample size that comprised over half a million people with T2D matched to at least one person of the same age, sex and ethnicity without diabetes. By studying a period prior to 2020, it assessed infection risks before the disruption of the COVID-19 pandemic, after which the relationship between BMI and COVID-19 infection risks became much more widely appreciated [1, 2]. The focus on adults with T2D had two advantages. Firstly, since they are generally seen regularly in UK primary care, it ensured a patient cohort with a high completeness of body mass index measurements (over 98%). Secondly, it allowed for an analysis of BMI values across a wide range, including appreciable numbers of participants with extremely high values (almost 25,000 people with T2D with a BMI > 43) which very few population studies have reported on.

The inclusion of the group without diabetes allowed a comparison of results across the same BMI categories, however they were less likely to have their weight measured recently, with only 75% of the originally matched cohort with an eligible BMI recorded during 2011–14. Although this resulted in unbalanced match-sets in the main analysis, the impact of this was minimal as the sensitivity analysis using 1:1 match-sets produced identical results. It might still be assumed that patients without diabetes and no BMI measurement are a healthier subset and might have more normal weights. However, the estimated prevalence of obesity (BMI ≥ 30) in the non-diabetes group with a recorded BMI (24%) was marginally lower than what was reported in the 2015 Health Survey for England (27%) [23]. A recent study investigating BMI and mortality using CPRD estimated a similar prevalence of obesity among adults with a measurement compared to national estimates [24].

Although the time since the last measurement will vary between patients, one might expect that sudden or significant weight changes are being captured, and using the last recorded BMI over a 4-year period may be a reasonable estimate of their BMI during the following 5-year follow-up period. Although the analysis could have used measurements from 2015 onwards to update a patient’s BMI this may increase the possibility of reverse causality where the infection had a resulted in a change in weight. Finally, it is important to acknowledge the potential limitation of BMI as a measure of clinical obesity [25], but despite this, BMI can still provide utility as a predictive measure of risk in large-scale epidemiological studies where it is routinely recorded.

### Comparisons with previous studies

The recent evidence base linking obesity to infection risk throughout the life course has been observed in several large epidemiological studies, mainly using BMI measurements in adult populations in developed countries [26]. The evidence regarding underweight is more sparse, although a 2013 systematic review concluded that being underweight was potentially a more serious risk factor for community acquitted pneumonia than obesity [27]. Instead, many studies have focused only on a presumed linear association between a range of infections and BMI. For example, a large Swedish cohort study of 39,163 adults found higher risk of many infections in participants with a BMI > 30 compared to normal weight (BMI = 18.5–25), but the study excluded underweight (BMI < 18.5) adults [8]. More recent studies from Denmark [9, 10] and Taiwan [11] have reported on the heterogenous shapes of the associations with different infections across a wider range of BMI values including underweight. Harpsøe et al [10], found that within the Danish National Birth Cohort (DNBC) of 75,001 middle age-women the relationship between any hospitalisation for infection over 12 years and their baseline BMI was U-shaped, with a particularly high risk among underweight women (BMI <18.5), largely driven by respiratory tract infections. The study of 101,447 adults in the Copenhagen General Population Study (CGPS) also found U-shaped associations between BMI and hospitalisation for any infection, in particular pneumonia [9]. Finally, a study of 120,864 adults in Taiwan showed U-shaped associations between BMI and hospitalisation for all infections, particularly LRT infections and septicaemia, and estimated that underweight people were at a greater risk than people who had obesity [11]. Our analysis similarly found (i) U-shape associations overall, particularly for hospitalisation related infections, which were not explained by smoking or co-morbidity, and (ii) these associations were strongest for both LRT infections and sepsis, which had the greatest relative risks among underweight diabetes and non-diabetes patients (BMI < 19) compared to their reference group. A recent study using the same CPRD dataset as utilised here but into 2020, found that among 584,854 people with T2D there was a higher risk of hospitalisation for COVID-19 among those with a BMI < 18.5 compared to 25–29.9, comparable to the more reported risks associated with a BMI of 40 or more [28].

A positive association between skin infections and obesity has been consistently observed [8–11, 29], with no evidence of an increase in risk among lower BMI levels [9–11, 29]. When the study from Copenhagen analysed their data using genetic instrumental variables, the only associations that remained significant from their initial analysis were with skin infections [9]. Obesity has been recognised as a predisposing factor for lower limb cellulitis, with a number of proposed mechanisms such as venous insufficiency, impaired lymphatic flow, increased skin fragility, and poorer hygiene [30]. A recent meta-analysis of case-control studies found that patients with cellulitis had 2.67 times odds of being classed as having obesity when compared with controls [31]. Our analysis was found that among all the infection types, the strongest positive association with increasing levels of BMI was with cellulitis. Similarly, in the Danish birth cohort study the risk of hospitalisation for erysipelas or cellulitis was five-fold higher among women with obesity (BMI ≥ 30) compared to normal levels of BMI [10].

We have previously reported on the increased risk of infection among people with diabetes and, due to the high prevalence of diabetes, estimated that almost 10% of all infection related hospitalisations among the adult population in England may be attributable to T2D [13]. Given the very high current prevalence of obesity, there is interest in understanding the relationship between these conditions. People living with either T2D or obesity share many of the same characteristics, and it has been suggested that the poor outcomes experienced by both groups may be due to an immune system dysfunction being triggered by chronic low-grade inflammation present in both T2D and obesity [15]. We found no evidence that diabetes mediated the relationship between BMI and infection risk. Although people with T2D were at higher risk of infections at every BMI category, their relative risk compared to a person with T2D and a BMI of 24–26 kg/m^2^ was very similar to that estimated among the non-diabetes group. Few population-based analyses have focused on people with T2D or compared the relationship with common infections and BMI to a non-diabetes population. A Taiwanese study of obesity, diabetes and tuberculosis found that the relationship was complex, such that participants who had obesity and diabetes had similar or lower risk of tuberculosis compared to participants without diabetes of normal weight [32].

### Implications

The current obesity epidemic has huge cost implications for healthcare and populations globally [33]. In the UK, it has been estimated that obesity accounts for approximately 3% of Gross Domestic Product [34]. Data from Australia has shown that among middle-aged adults, there is a strong dose response relationship between BMI and hospital costs and length of stay [35]. Similarly, rising BMI is inversely related to health-related quality of life measures [33]. While the health consequences of obesity predominately focus on non-communicable disease, our study suggests infections may represent another important consequence to consider. The association with a wide burden of disease, has led to the suggestion that obesity could be an “amenable target for complex multimorbidity prevention” [29]. A recent retrospective cohort study in the UK found that higher BMI categories were associated with increased healthcare expenditure, particularly when obesity-related complications were present [36]. The introduction of GLP-1 agonists as novel weight loss therapies, such as semaglutide, may have significant impact on population trends in obesity, and could represent a turnaround in the effectiveness of weight loss public health strategies [37]. With trial evidence additionally suggesting lower rates of infection while on semaglutide [38], their increased usage could play a part in reducing infection risk in the population, as well as the non-infectious consequences of obesity. However, the roll out of these weight-loss drugs is unlikely to be fast and may increase inequities that already exist regarding obesity in the population [39]. Although a recent study using CPRD data found that while all-cause mortality rates had significantly declined in people with obesity (BMI ≥ 30) from 2004-2019, no significant reduction was seen for infection specific deaths [24]. Therefore, more targeted infection prevention strategies in higher risk patients based on their BMI such as appropriate vaccination, and increased patient and clinician awareness of the need for early assessment of infections, could result in prompter diagnosis and effective treatment, with the potential to reduce infection-related hospitalisations.

## Conclusions

In conclusion, our study has emphasised the impact of obesity on infection risk, and that this relationship appears to act independently of Type 2 diabetes. In addition, some infections such as pneumonia and sepsis are more common in underweight people and are not simply explained by other health conditions.

## Supplementary information

Supplementary Information

## Data Availability

The study is based on electronic health data from the Clinical Practice Research Datalink (CPRD) obtained under license from the UK Medicines and Healthcare Products Regulatory Agency (MHRA). CPRD data governance and the license to use CPRD data does not allow distribution of patient data directly to other parties. Researchers must apply directly to CPRD for data access (https://www.cprd.com).
