# Supplementary material for: Body mass index and infection risks in people with and without type 2 diabetes: A cohort study using electronic health records
Source: Int J Obes (Lond). 2025 Jul 18;49(9):1800–9. doi: 10.1038/s41366-025-01828-z (PMC12463668; doi:10.1038/s41366-025-01828-z)
Supplement: Supplementary file 1 — Supplementary Information [file 41366_2025_1828_MOESM1_ESM.docx]

Supplementary Information

Contents

[Table S1: Adjusted incidence rate ratios for infections during 2015-19 by BMI in people with T2D and without diabetes, by sex 2](#_Toc187229066)

[Table S2: Attributable risk fraction estimates for infections during 2015-19 using a reference BMI category of 24-26 kg/m^2^ people with T2D and without diabetes 3](#_Toc187229067)

[Table S3: Impact of adjustment and using 1:1 match-sets on incidence rate ratios for infections during 2015-19 by body mass index in people with T2D and without diabetes 4](#_Toc187229068)

[Table S4: Adjusted incidence rate ratios for infections during 2015-19 by BMI in people with T2D and w/o diabetes, NEW sensitivity 5](#_Toc187229069)

[Figure S1: Summary of selected study participants with type 2 diabetes, and matched group without any diabetes 6](#_Toc187229070)

[Figure S2: Distribution of BMI by age in people with T2D and without diabetes 7](#_Toc187229071)

[Figure S3: Mean age in 2015 by BMI categories in people with T2D and without diabetes, overall and by sex 8](#_Toc187229072)

[Figure S4: Incidence rates for infections during 2015-19 by BMI categories in people with T2D and without diabetes, overall and by sex 9](#_Toc187229073)

[Figure S5: Adjusted incidence rate ratios for infections during 2015-19 by BMI in people with T2D and without diabetes, by age (3 groups) 10](#_Toc187229074)

## Table S1: Adjusted incidence rate ratios for infections during 2015-19 by BMI in people with T2D and without diabetes, by sex

| BMI (kg/m^2^) | Type 2 Diabetes (n=516,935) | | Non-Diabetes (n=751,909) | |
| --- | --- | --- | --- | --- |
|  | Women | Men | Women | Men |
|  | IRR (95%CI) | IRR (95%CI) | IRR (95%CI) | IRR (95%CI) |
| Primary Care |  |  |  |  |
| <19 | 1.07 (1.02-1.11) | 1.14 (1.06-1.22) | 1.07 (1.05-1.09) | 1.21 (1.17-1.25) |
| 19-22 | 0.97 (0.95-1.00) | 1.04 (1.01-1.07) | 0.97 (0.95-0.98) | 1.05 (1.03-1.06) |
| 22-24 | 0.96 (0.94-0.98) | 0.97 (0.95-1.00) | 0.96 (0.95-0.98) | 1.00 (0.99-1.01) |
| 24-26 | 1 (Reference) | 1 (Reference) | 1 (Reference) | 1 (Reference) |
| 26-28 | 1.03 (1.01-1.05) | 1.02 (1.01-1.04) | 1.03 (1.02-1.05) | 1.05 (1.04-1.06) |
| 28-30 | 1.08 (1.07-1.10) | 1.08 (1.06-1.10) | 1.09 (1.07-1.10) | 1.10 (1.08-1.11) |
| 30-32 | 1.12 (1.10-1.14) | 1.15 (1.13-1.17) | 1.11 (1.10-1.13) | 1.16 (1.14-1.18) |
| 32-35 | 1.17 (1.15-1.19) | 1.24 (1.22-1.26) | 1.16 (1.14-1.18) | 1.26 (1.24-1.28) |
| 35-39 | 1.27 (1.24-1.29) | 1.37 (1.34-1.39) | 1.25 (1.22-1.27) | 1.37 (1.34-1.40) |
| 39-43 | 1.33 (1.31-1.36) | 1.54 (1.50-1.57) | 1.31 (1.28-1.34) | 1.52 (1.47-1.58) |
| 43-48 | 1.48 (1.45-1.51) | 1.69 (1.64-1.74) | 1.41 (1.36-1.46) | 1.73 (1.64-1.83) |
| 48- | 1.59 (1.55-1.64) | 2.03 (1.96-2.11) | 1.56 (1.49-1.64) | 1.91 (1.75-2.08) |
|  |  |  |  |  |
| Hospitalisations |  |  |  |  |
| <19 | 1.83 (1.71-1.95) | 2.23 (2.05-2.41) | 1.87 (1.80-1.95) | 2.53 (2.43-2.64) |
| 19-22 | 1.29 (1.24-1.35) | 1.37 (1.31-1.42) | 1.23 (1.20-1.27) | 1.51 (1.47-1.55) |
| 22-24 | 1.10 (1.06-1.15) | 1.11 (1.07-1.15) | 1.03 (0.99-1.06) | 1.17 (1.14-1.20) |
| 24-26 | 1 (Reference) | 1 (Reference) | 1 (Reference) | 1 (Reference) |
| 26-28 | 1.01 (0.97-1.04) | 0.98 (0.95-1.01) | 1.02 (0.99-1.05) | 0.96 (0.94-0.99) |
| 28-30 | 1.02 (0.99-1.06) | 1.00 (0.97-1.03) | 1.05 (1.02-1.09) | 1.02 (0.99-1.04) |
| 30-32 | 1.09 (1.06-1.13) | 1.05 (1.02-1.09) | 1.11 (1.07-1.15) | 1.09 (1.06-1.12) |
| 32-35 | 1.15 (1.11-1.19) | 1.16 (1.13-1.19) | 1.21 (1.17-1.26) | 1.21 (1.18-1.25) |
| 35-39 | 1.30 (1.26-1.35) | 1.30 (1.26-1.35) | 1.37 (1.32-1.43) | 1.35 (1.30-1.41) |
| 39-43 | 1.49 (1.43-1.55) | 1.54 (1.49-1.60) | 1.60 (1.51-1.69) | 1.72 (1.61-1.83) |
| 43-48 | 1.71 (1.63-1.78) | 1.86 (1.77-1.95) | 1.93 (1.79-2.08) | 1.81 (1.62-2.02) |
| 48- | 2.26 (2.15-2.37) | 2.43 (2.28-2.59) | 2.39 (2.15-2.66) | 2.57 (2.19-3.03) |

Separate models for T2D and non-diabetes. Incidence rate ratios (IRRs) adjust for age, sex, ethnicity, deprivation, smoking and co-morbidity count.

## Table S2: Attributable risk fraction estimates for infections during 2015-19 using a reference BMI category of 24-26 kg/m^2^ people with T2D and without diabetes

|  | Type 2 Diabetes (n=516,935) | | Non-Diabetes (n=751,909) | |
| --- | --- | --- | --- | --- |
|  | Primary Care | Hospitalisations | Primary Care | Hospitalisations |
|  |  |  |  |  |
| All | 11.4% | 11.5% | 5.9% | 10.0% |
|  |  |  |  |  |
| Ages 18-50 | 18.1% | 13.0% | 4.7% | 13.0% |
| Ages 51-70 | 14.2% | 14.2% | 6.5% | 11.0% |
| Ages 71-90 | 7.5% | 9.0% | 5.7% | 9.4% |
|  |  |  |  |  |
| Women | 11.5% | 14.1% | 4.6% | 10.7% |
| Men | 11.9% | 9.8% | 7.4% | 9.0% |

- Derived from separate models for T2D and non-diabetes which estimated IRRs for different BMI categories compared to a reference category of 24-26 kg/m^2^, adjusted for age, sex, ethnicity, deprivation, smoking & co-morbidity count (Table 3).
- The attributable risk fractions are estimated in each BMI category by “% in BMI category” x [(IRR-1) / IRR] and then summed across all BMI categories to obtain overall estimate.

## Table S3: Impact of adjustment and using 1:1 match-sets on incidence rate ratios for infections during 2015-19 by body mass index in people with T2D and without diabetes

| BMI (kg/m^2^) | Type 2 Diabetes | | | Non-Diabetes | | |
| --- | --- | --- | --- | --- | --- | --- |
|  | All (n=516,935) | All (n=516,935) | 1:1 match-sets (n=471,474) | All (n=751,909) | All (n=751,909) | 1:1 match-sets (n=471,474) |
|  | IRR1 (95%CI) | IRR2 (95%CI) | IRR2 (95%CI) | IRR1 (95%CI) | IRR2 (95%CI) | IRR2 (95%CI) |
| Primary Care |  |  |  |  |  |  |
| <19 | 1.09 (1.05-1.13) | 1.08 (1.04-1.12) | 1.07 (1.03-1.11) | 1.13 (1.11-1.15) | 1.11 (1.09-1.13) | 1.11 (1.09-1.14) |
| 19-22 | 1.00 (0.98-1.02) | 1.00 (0.98-1.01) | 0.99 (0.98-1.01) | 1.00 (0.99-1.01) | 1.00 (0.99-1.01) | 1.00 (0.99-1.02) |
| 22-24 | 0.97 (0.96-0.99) | 0.97 (0.95-0.98) | 0.97 (0.96-0.99) | 0.98 (0.97-0.99) | 0.98 (0.97-0.99) | 0.99 (0.97-1.00) |
| 24-26 | 1 (Reference) | 1 (Reference) | 1 (Reference) | 1 (Reference) | 1 (Reference) | 1 (Reference) |
| 26-28 | 1.02 (1.01-1.04) | 1.02 (1.01-1.04) | 1.03 (1.01-1.04) | 1.05 (1.04-1.06) | 1.04 (1.03-1.05) | 1.04 (1.03-1.05) |
| 28-30 | 1.08 (1.07-1.09) | 1.08 (1.06-1.09) | 1.08 (1.07-1.09) | 1.10 (1.09-1.11) | 1.09 (1.08-1.10) | 1.09 (1.08-1.11) |
| 30-32 | 1.13 (1.12-1.15) | 1.13 (1.11-1.14) | 1.13 (1.12-1.15) | 1.15 (1.14-1.17) | 1.13 (1.12-1.14) | 1.13 (1.12-1.15) |
| 32-35 | 1.21 (1.19-1.22) | 1.20 (1.18-1.21) | 1.20 (1.19-1.22) | 1.23 (1.22-1.25) | 1.20 (1.19-1.21) | 1.21 (1.19-1.22) |
| 35-39 | 1.32 (1.30-1.34) | 1.30 (1.29-1.32) | 1.31 (1.29-1.33) | 1.33 (1.32-1.35) | 1.29 (1.28-1.31) | 1.30 (1.28-1.32) |
| 39-43 | 1.43 (1.41-1.45) | 1.41 (1.39-1.43) | 1.41 (1.39-1.43) | 1.44 (1.41-1.47) | 1.39 (1.36-1.41) | 1.39 (1.36-1.43) |
| 43-48 | 1.59 (1.57-1.62) | 1.57 (1.54-1.59) | 1.56 (1.53-1.59) | 1.58 (1.53-1.62) | 1.51 (1.47-1.56) | 1.49 (1.43-1.54) |
| 48- | 1.78 (1.75-1.82) | 1.75 (1.71-1.78) | 1.75 (1.72-1.79) | 1.74 (1.67-1.82) | 1.68 (1.61-1.75) | 1.68 (1.59-1.77) |
|  |  |  |  |  |  |  |
| Hospitalisations |  |  |  |  |  |  |
| <19 | 2.01 (1.91-2.11) | 1.91 (1.83-2.02) | 1.93 (1.83-2.03) | 2.30 (2.24-2.37) | 2.11 (2.05-2.17) | 2.08 (2.01-2.16) |
| 19-22 | 1.32 (1.28-1.36) | 1.31 (1.28-1.35) | 1.30 (1.26-1.34) | 1.39 (1.36-1.42) | 1.36 (1.34-1.39) | 1.36 (1.32-1.38) |
| 22-24 | 1.10 (1.08-1.13) | 1.10 (1.08-1.13) | 1.10 (1.07-1.13) | 1.10 (1.08-1.13) | 1.11 (1.09-1.13) | 1.11 (1.08-1.14) |
| 24-26 | 1 (Reference) | 1 (Reference) | 1 (Reference) | 1 (Reference) | 1 (Reference) | 1 (Reference) |
| 26-28 | 1.01 (0.98-1.03) | 0.99 (0.97-1.01) | 0.99 (0.96-1.01) | 1.00 (0.98-1.02) | 0.98 (0.96-1.00) | 0.98 (0.96-1.00) |
| 28-30 | 1.04 (1.01-1.06) | 1.00 (0.98-1.03) | 1.00 (0.98-1.03) | 1.07 (1.05-1.09) | 1.03 (1.01-1.05) | 1.03 (1.01-1.06) |
| 30-32 | 1.12 (1.09-1.14) | 1.07 (1.04-1.09) | 1.07 (1.04-1.09) | 1.16 (1.13-1.18) | 1.09 (1.07-1.12) | 1.08 (1.05-1.11) |
| 32-35 | 1.23 (1.20-1.25) | 1.15 (1.13-1.18) | 1.15 (1.12-1.17) | 1.31 (1.28-1.34) | 1.22 (1.19-1.24) | 1.21 (1.17-1.24) |
| 35-39 | 1.41 (1.38-1.44) | 1.30 (1.27-1.33) | 1.29 (1.26-1.32) | 1.52 (1.47-1.56) | 1.37 (1.34-1.41) | 1.37 (1.32-1.42) |
| 39-43 | 1.66 (1.62-1.71) | 1.51 (1.47-1.56) | 1.51 (1.47-1.55) | 1.87 (1.79-1.95) | 1.66 (1.60-1.74) | 1.60 (1.52-1.69) |
| 43-48 | 1.97 (1.91-2.04) | 1.77 (1.72-1.83) | 1.76 (1.70-1.82) | 2.19 (2.06-2.33) | 1.93 (1.82-2,05) | 1.81 (1.67-1.96) |
| 48- | 2.65 (2.55-2.75) | 2.35 (2.26-2.44) | 2.35 (2.26-2.45) | 2.85 (2.61-3.12) | 2.52 (2.30-2.75) | 2.43 (2.17-2.72) |

Separate models for T2D and non-diabetes. Incidence rate ratios: IRR1 = adjusts for age & sex. IRR2 = adjusts for age, sex, ethnicity, deprivation, smoking & co-morbidity.

## Table S4: Adjusted incidence rate ratios for infections during 2015-19 by BMI in people with T2D and w/o diabetes, NEW sensitivity

| BMI (kg/m^2^) | Type 2 Diabetes (n=516,935) | | | Non-Diabetes (n=751,909) | | |
| --- | --- | --- | --- | --- | --- | --- |
|  | Never smokers (n=193,200) | No other chronic disease (n=144,204) | Never smokers + no other chronic disease (n=59,914) | Never smokers (n=329,071) | No other chronic disease (n=397,164) | Never smokers + no other chronic disease (n=189,087) |
|  | IRR (95%CI) | IRR (95%CI) | IRR (95%CI) | IRR (95%CI) | IRR (95%CI) | IRR (95%CI) |
| Primary Care |  |  |  |  |  |  |
| <19 | 1.04 (0.97-1.10) | 0.99 (0.92-1.06) | 0.93 (0.83-1.05) | 1.13 (1.09-1.16) | 1.06 (1.03-1.09) | 1.05 (1.01-1.09) |
| 19-22 | 0.98 (0.95-1.01) | 0.94 (0.91-0.98) | 0.91 (0.87-0.96) | 0.99 (0.98-1.01) | 0.97 (0.96-0.99) | 0.97 (0.94-0.99) |
| 22-24 | 0.96 (0.93-0.98) | 0.94 (0.91-0.97) | 0.90 (0.86-0.94) | 0.99 (0.97-1.00) | 0.97 (0.95-0.98) | 0.96 (0.94-0.98) |
| 24-26 | 1.00 (Reference) | 1.00 (Reference) | 1.00 (Reference) | 1.00 (Reference) | 1.00 (Reference) | 1.00 (Reference) |
| 26-28 | 1.01 (0.99-1.03) | 1.05 (1.03-1.08) | 1.02 (0.98-1.06) | 1.06 (1.04-1.07) | 1.04 (1.03-1.06) | 1.04 (1.02-1.07) |
| 28-30 | 1.08 (1.06-1.10) | 1.11 (1.09-1.14) | 1.09 (1.05-1.13) | 1.12 (1.10-1.14) | 1.11 (1.09-1.13) | 1.13 (1.11-1.16) |
| 30-32 | 1.12 (1.10-1.14) | 1.18 (1.15-1.21) | 1.16 (1.12-1.21) | 1.15 (1.13-1.17) | 1.14 (1.12-1.16) | 1.15 (1.12-1.18) |
| 32-35 | 1.18 (1.16-1.20) | 1.24 (1.21-1.27) | 1.21 (1.17-1.26) | 1.22 (1.20-1.24) | 1.22 (1.20-1.24) | 1.22 (1.19-1.26) |
| 35-39 | 1.29 (1.26-1.31) | 1.37 (1.34-1.40) | 1.33 (1.28-1.38) | 1.31 (1.28-1.34) | 1.34 (1.31-1.37) | 1.31 (1.27-1.35) |
| 39-43 | 1.41 (1.37-1.44) | 1.49 (1.45-1.53) | 1.43 (1.37-1.49) | 1.40 (1.35-1.44) | 1.46 (1.41-1.51) | 1.43 (1.37-1.51) |
| 43-48 | 1.56 (1.52-1.61) | 1.61 (1.56-1.66) | 1.58 (1.50-1.66) | 1.50 (1.43-1.56) | 1.55 (1.48-1.63) | 1.46 (1.36-1.57) |
| 48- | 1.74 (1.68-1.80) | 1.78 (1.71-1.85) | 1.71 (1.60-1.82) | 1.62 (1.51-1.73) | 1.73 (1.62-1.85) | 1.61 (1.45-1.79) |
|  |  |  |  |  |  |  |
| Hospitalisations |  |  |  |  |  |  |
| <19 | 1.83 (1.67-2.01) | 2.19 (1.98-2.43) | 2.01 (1.66-2.44) | 1.73 (1.64-1.83) | 2.22 (2.12-2.32) | 1.71 (1.57-1.87) |
| 19-22 | 1.26 (1.19-1.32) | 1.36 (1.27-1.46) | 1.27 (1.13-1.43) | 1.21 (1.16-1.25) | 1.32 (1.28-1.37) | 1.11 (1.05-1.18) |
| 22-24 | 1.12 (1.07-1.17) | 1.11 (1.04-1.18) | 1.10 (0.99-1.22) | 1.06 (1.03-1.10) | 1.05 (1.02-1.09) | 1.00 (0.94-1.06) |
| 24-26 | 1.00 (Reference) | 1.00 (Reference) | 1.00 (Reference) | 1.00 (Reference) | 1.00 (Reference) | 1.00 (Reference) |
| 26-28 | 1.03 (0.99-1.07) | 1.07 (1.02-1.13) | 1.07 (0.98-1.18) | 1.05 (1.01-1.09) | 1.02 (0.98-1.05) | 1.07 (1.01-1.13) |
| 28-30 | 1.03 (0.99-1.07) | 1.09 (1.03-1.15) | 1.09 (0.99-1.20) | 1.10 (1.06-1.14) | 1.10 (1.06-1.14) | 1.17 (1.10-1.24) |
| 30-32 | 1.11 (1.07-1.16) | 1.16 (1.10-1.23) | 1.16 (1.05-1.27) | 1.18 (1.13-1.23) | 1.19 (1.14-1.24) | 1.26 (1.18-1.35) |
| 32-35 | 1.25 (1.20-1.30) | 1.25 (1.19-1.32) | 1.34 (1.22-1.47) | 1.28 (1.22-1.33) | 1.35 (1.30-1.41) | 1.37 (1.28-1.48) |
| 35-39 | 1.41 (1.35-1.47) | 1.46 (1.39-1.55) | 1.54 (1.39-1.69) | 1.49 (1.42-1.57) | 1.55 (1.47-1.63) | 1.66 (1.52-1.81) |
| 39-43 | 1.61 (1.53-1.69) | 1.68 (1.58-1.79) | 1.79 (1.59-2.01) | 1.84 (1.72-1.97) | 1.80 (1.66-1.95) | 1.99 (1.75-2.26) |
| 43-48 | 1.99 (1.88-2.11) | 1.85 (1.71-2.00) | 2.05 (1.80-2.34) | 2.33 (2.11-2.56) | 2.14 (1.91-2.41) | 2.44 (2.03-2.92) |
| 48- | 2.71 (2.54-2.89) | 2.63 (2.42-2.86) | 2.80 (2.42-3.24) | 2.61 (2.25-3.02) | 2.61 (2.23-3.05) | 2.50 (1.90-3.29) |

Separate models for T2D and non-diabetes. Incidence rate ratios (IRRs) adjust for age, sex, ethnicity, deprivation, smoking & co-morbidity count.

## Figure S1: Summary of selected study participants with type 2 diabetes, and matched group without any diabetes

**527,151 (6.0%)**
with Type 2 diabetes diagnosed prior to 2015*

**8,722,348 (100%)**Patients aged 18-90 active on CPRD on 1^st^ January 2015 and registered for > 1 year

**516,935 (98.3%)**with a BMI measured in 2011-14

**525,812**with at least 1 match to non-diabetes person

**1,008,898**without diabetes or prediabetes matched on age, sex and ethnicity

**471,474 (89.7%)**1:1 matched to a non-diabetes person with a BMI in 2011-14

**471,474**1:1 matched to a Type 2 Diabetes person with a BMI in 2011-14

**751,909 (74.5%)**with a BMI measured in 2011-14

Main analysis

Sensitivity analysis

CPRD = Clinical Practice Research Datalink. BMI = Body Mass Index.

*Read code lists for diabetes are available in the repository <https://doi.org/10.24376/rd.sgul.21565557.v1>

## Figure S2: Distribution of BMI by age in people with T2D and without diabetes

(a) Age 18-50 years


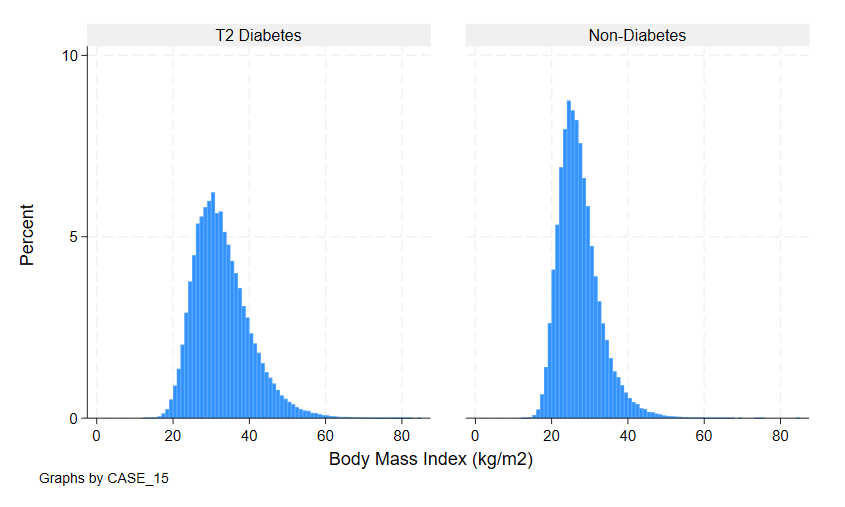


(b) Age 51-70 years


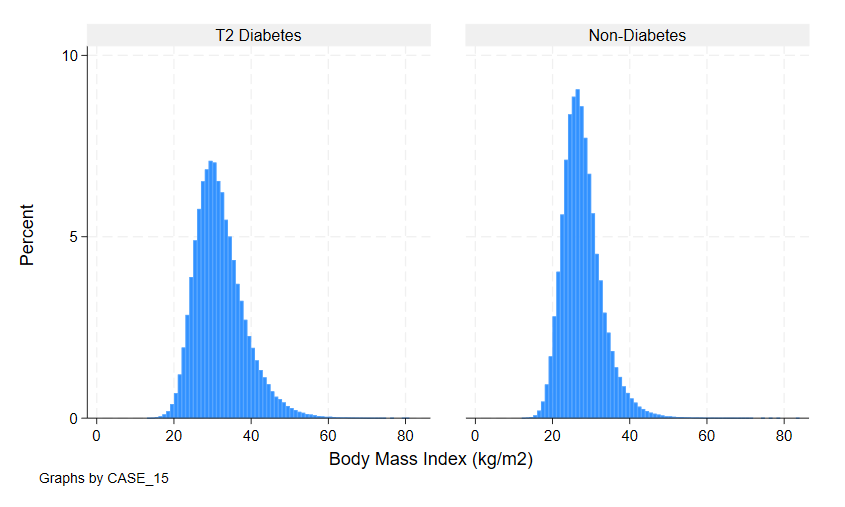


(c) Age 71-90 years


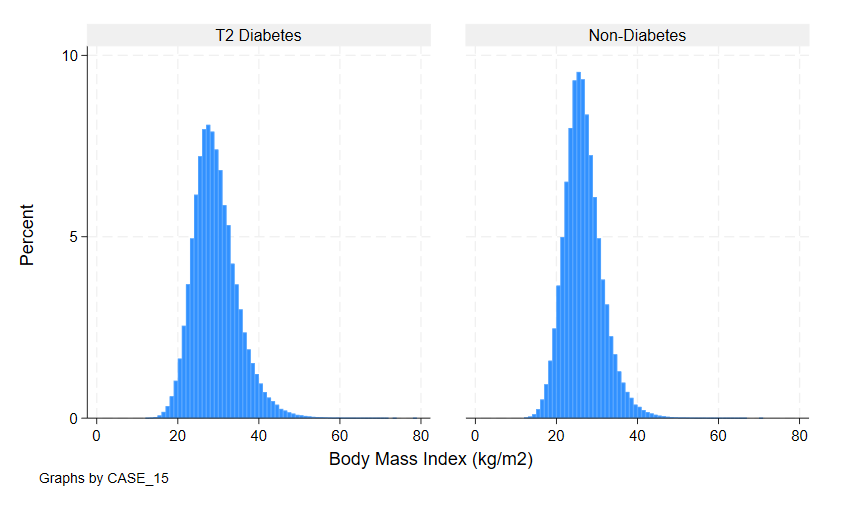


## Figure S3: Mean age in 2015 by BMI categories in people with T2D and without diabetes, overall and by sex


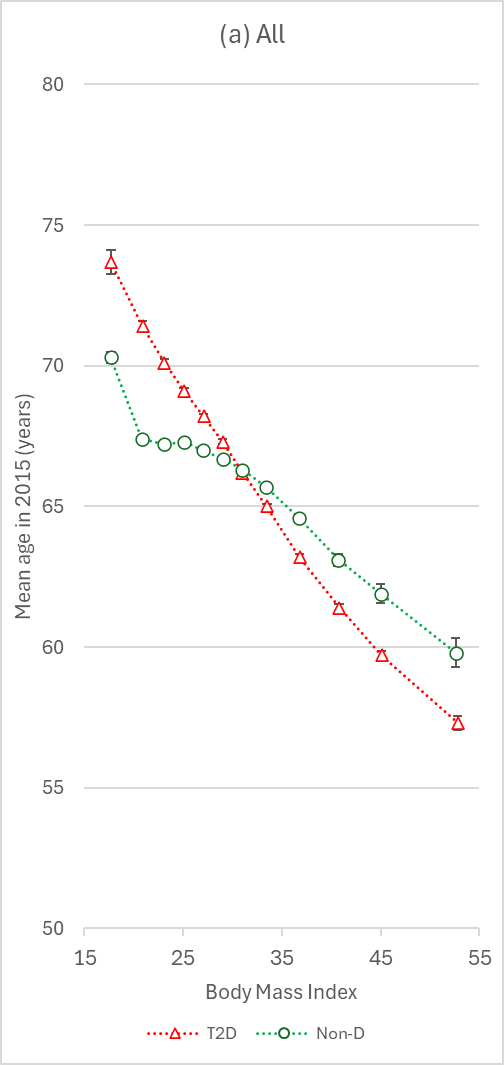

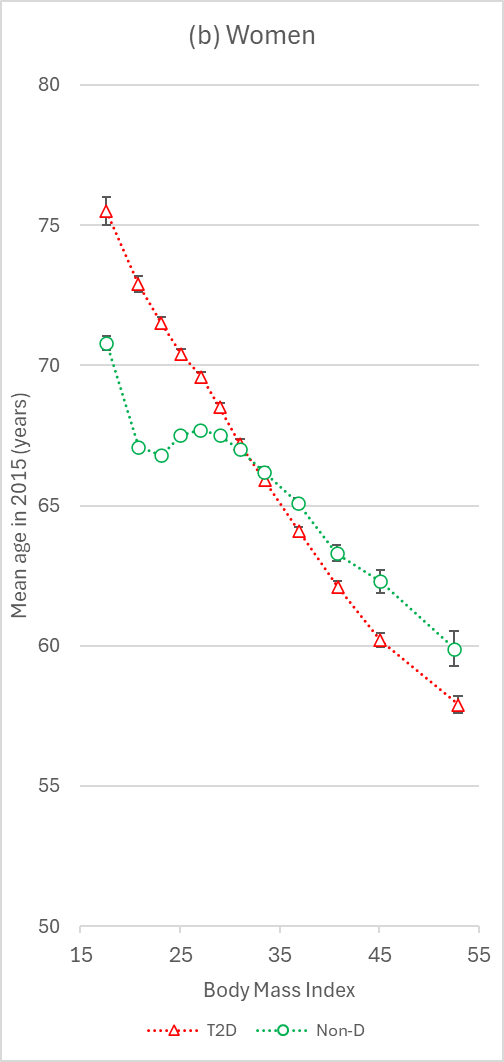

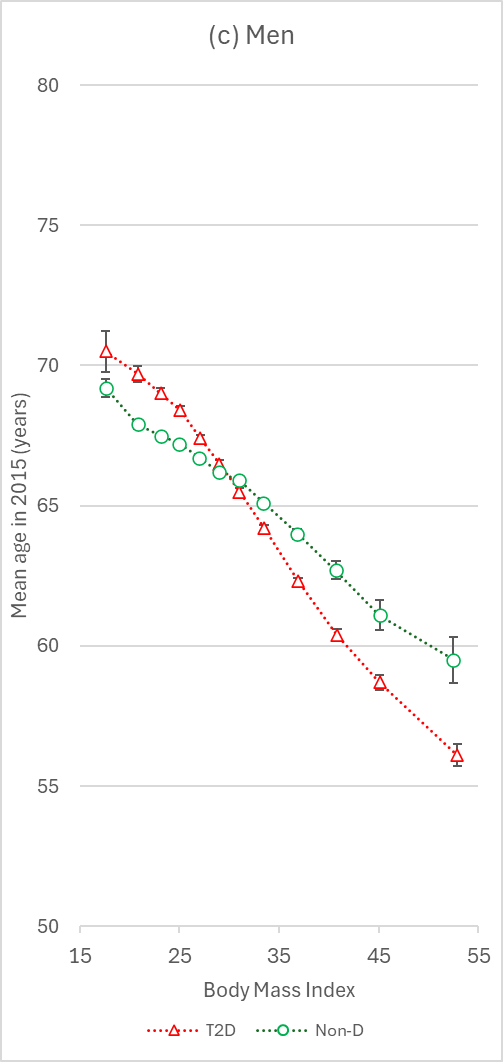


## Figure S4: Incidence rates for infections during 2015-19 by BMI categories in people with T2D and without diabetes, overall and by sex


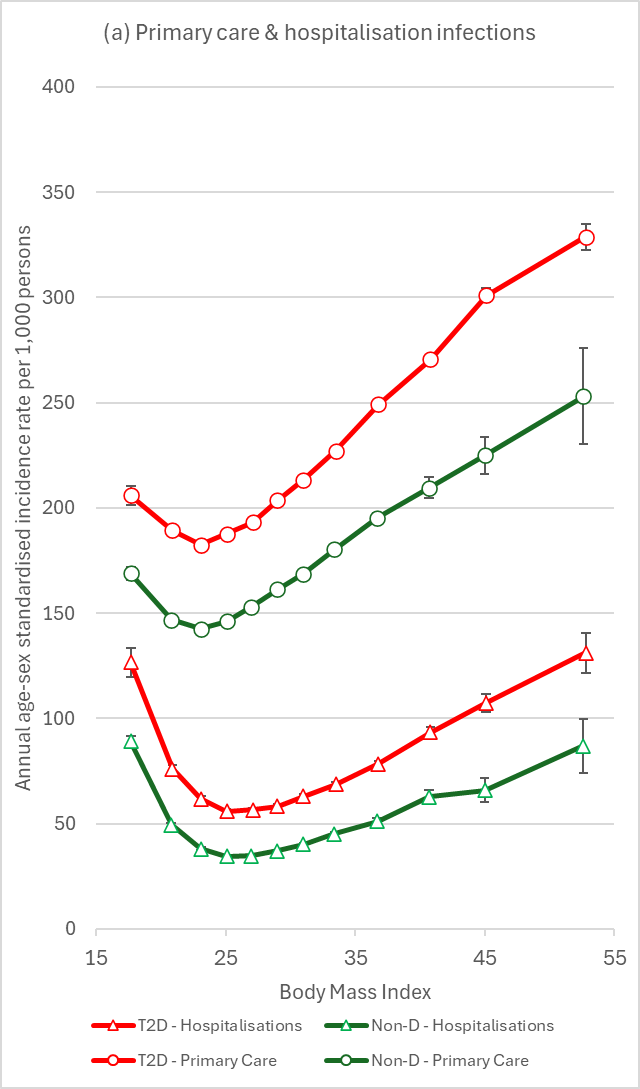

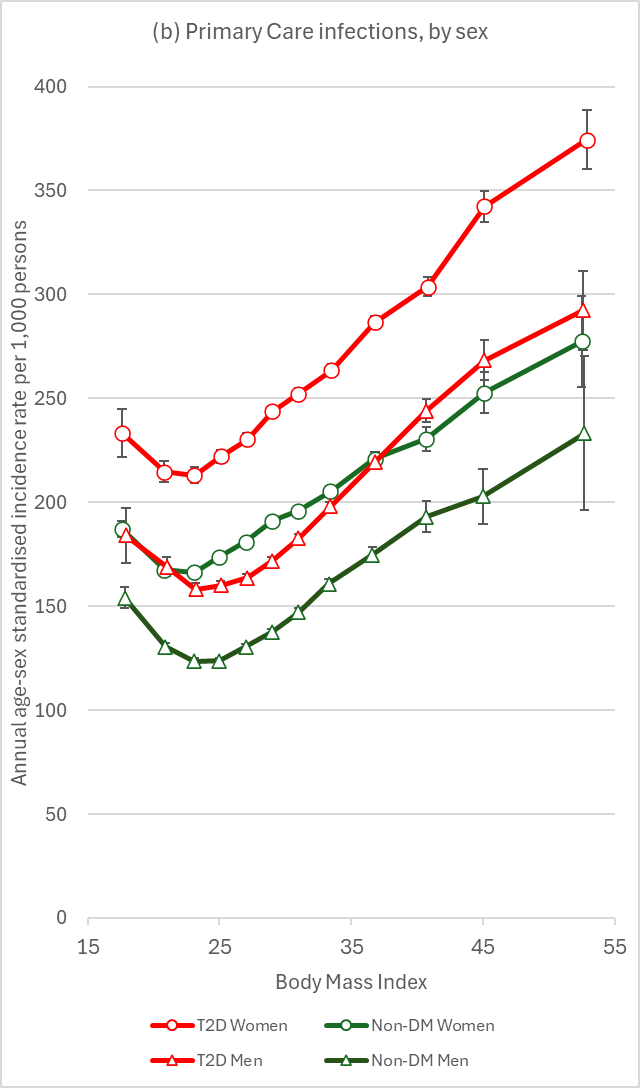

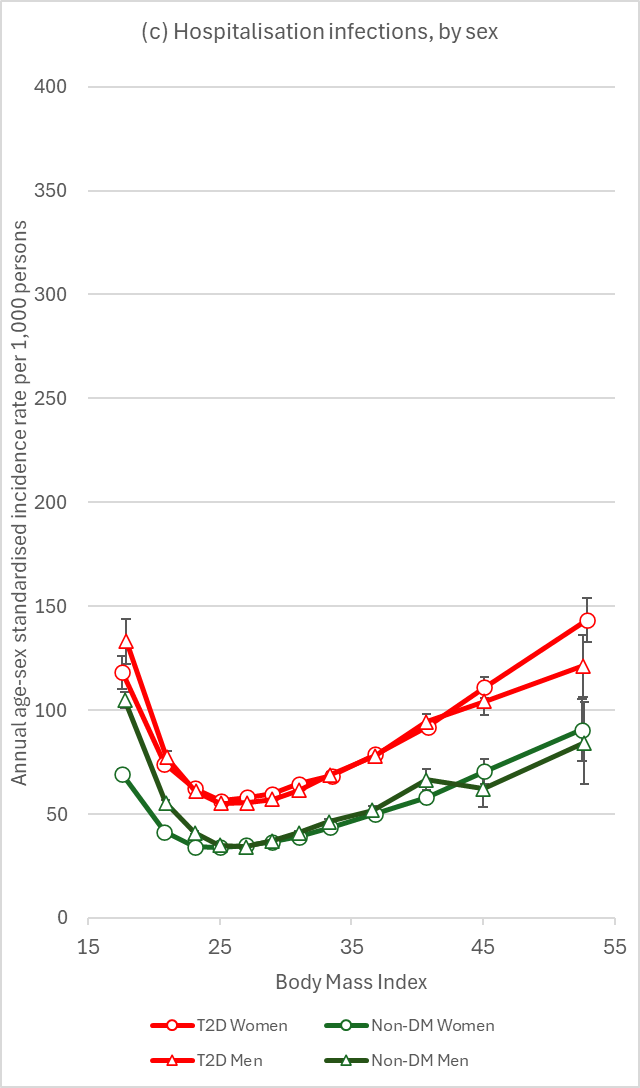


Note: Incidence rates are age-sex standardised to the comparator distribution of patients with T2D.

## Figure S5: Adjusted incidence rate ratios for infections during 2015-19 by BMI in people with T2D and without diabetes, by age (3 groups)


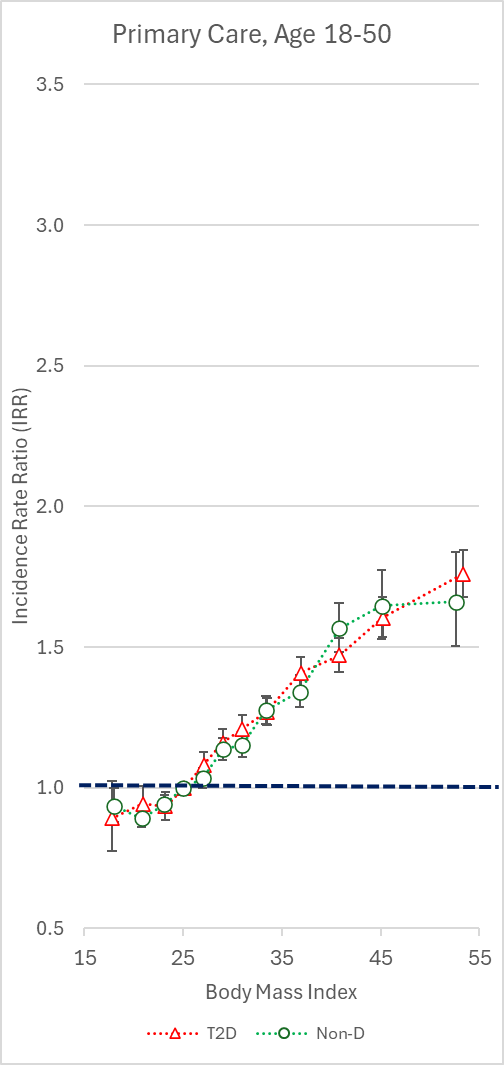

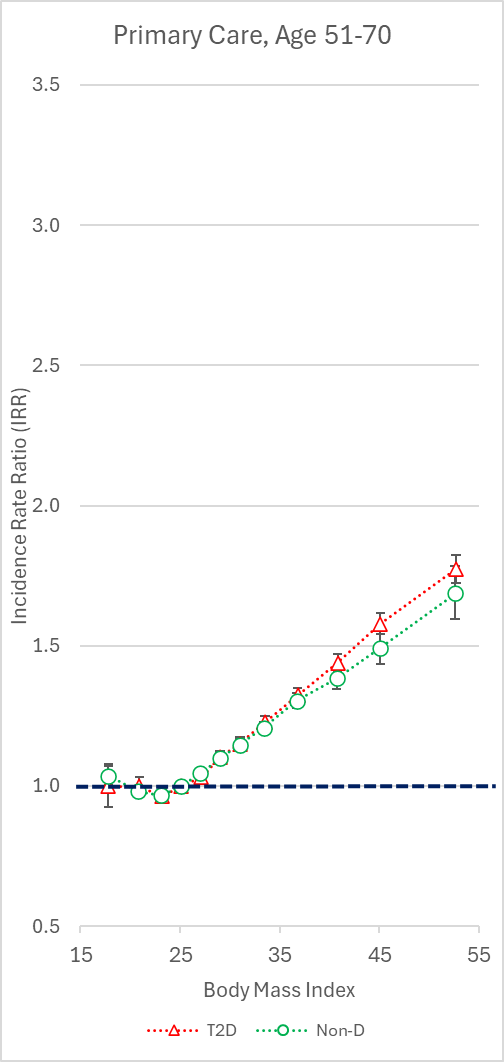

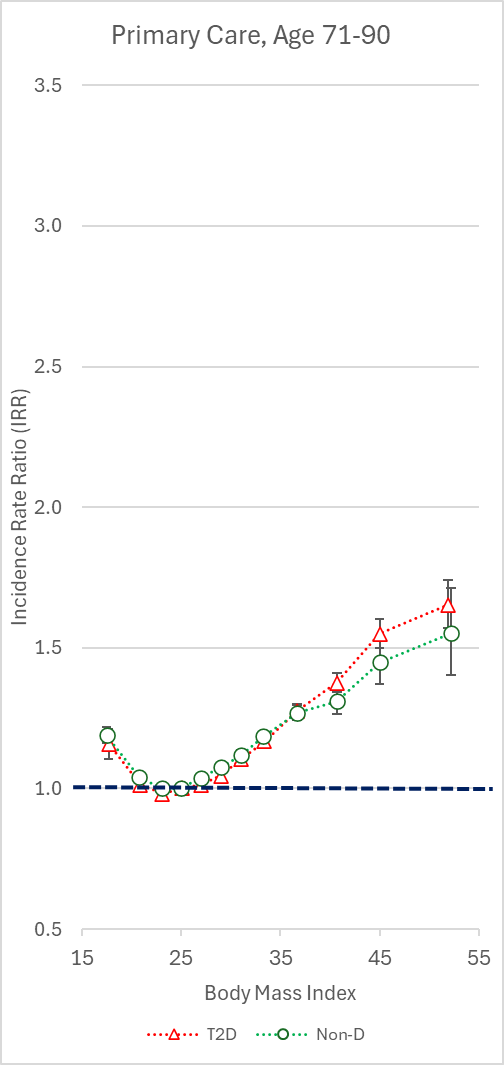

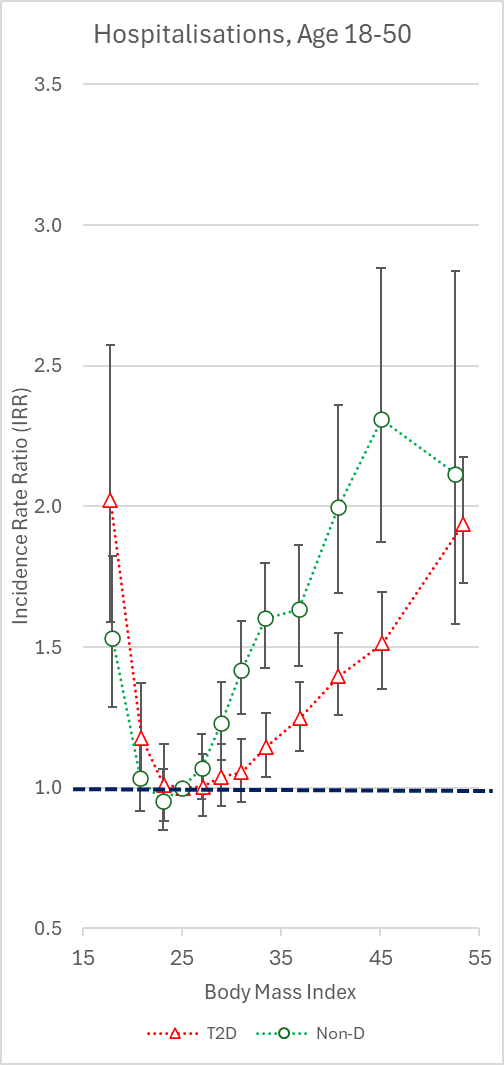

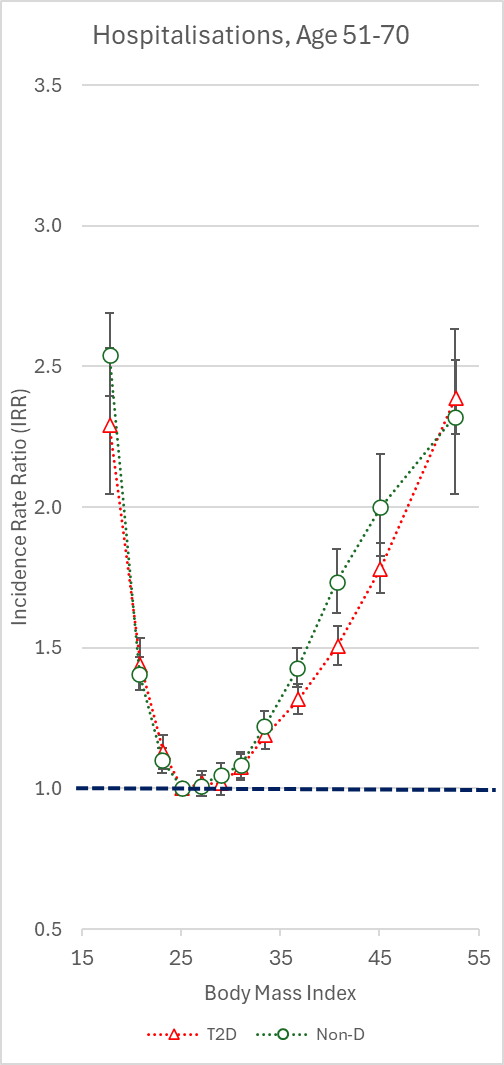

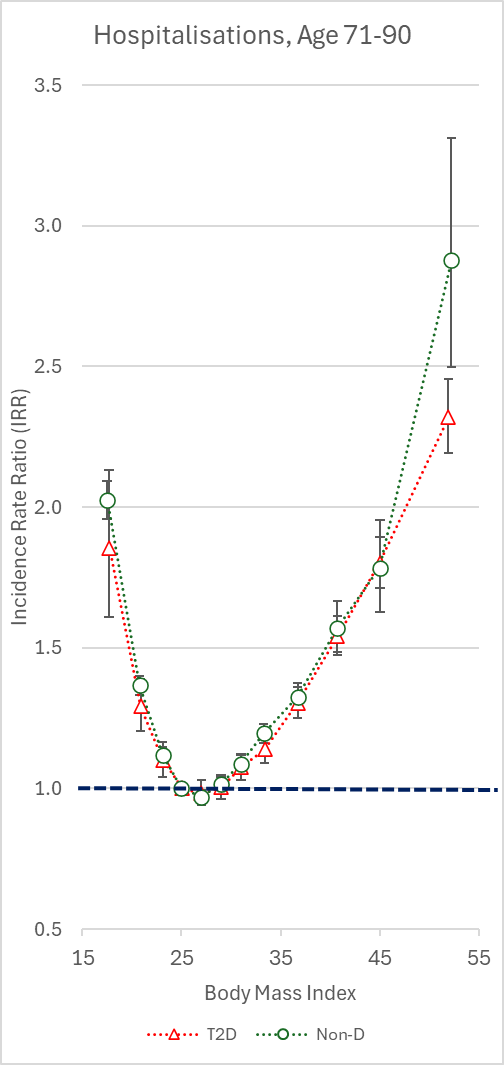


Separate models for T2D and non-diabetes adjusting for age, sex, ethnicity, deprivation, smoking and co-morbidity count.
